# European Network for Optimization of Veterinary Antimicrobial Therapy (ENOVAT) guidelines for antimicrobial and non-steroidal anti-inflammatory drug use in bovine respiratory disease

**DOI:** 10.12688/openreseurope.24099.1

**Published:** 2026-08-14

**Authors:** Karolina Scahill, Sébastien Buczinski, Clair L. Firth, Bart Pardon, Maria Stokstad, Amelia R. Woolums, Ramazan Yildiz, Lise Marie Ånestad, Mickael Cargnel, Luis Pedro Carmo, Pierre-Louis Toutain, Luca Guardabassi

**Affiliations:** 1 International Veterinary Evidence-based Guidelines Centre, Department of Veterinary Clinical Science, University of Copenhagen, Frederiksberg C, Denmark; 2 Infectious disease, The University of Edinburgh College of Medicine and Veterinary Medicine,, Edinburgh, UK; 3 Département des sciences cliniques, Faculté de médecine vétérinaire, Université de Montréal, Quebec, Canada; 4 Centre for Veterinary Public Health & One Health, Clinical Department for Farm Animals and Food System Transformation, University of Veterinary Medicine Vienna, Vienna, Austria; 5 U Gent Calf Health, Department of Internal Medicine, Reproduction and Population Medicine, Faculty of Veterinary Medicine, Ghent University, Merelbeke, Belgium; 6 Department of Production Animal Clinical Sciences, Faculty of Veterinary Medicine, Norwegian University of Life Sciences, Ås, Norway; 7 Department of Pathobiology and Population Medicine, Mississippi State University, Mississippi, USA; 8 Department of Internal Medicine, Faculty of Veterinary Medicine,, Mehmet Akif Ersoy University, Burdur, Turkey; 9 Norwegian Veterinary Institute, Ås, Norway; 10 Department of Infectious Diseases in Animals, Sciensano, Brussels, Belgium; 11 Veterinary Public Health Institute, Vetsuisse Faculty, University of Bern, Bern, Switzerland; 12 Comparative Biomedical Sciences, The Royal Veterinary College, London, UK; 13 Department of Veterinary and Animal Sciences, University of Copenhagen, Frederiksberg C, Denmark

**Keywords:** Bovine respiratory disease (BRD), Antimicrobial therapy, Antimicrobial stewardship, Non-steroidal anti-inflammatory drugs (NSAIDs), Mycoplasmopsis bovis, Evidence-based guidelines, GRADE, Evidence-to-Decision (EtD)

## Abstract

**Background:**

Bovine respiratory disease (BRD) is a major cause of morbidity, mortality, impaired welfare, production loss, and antimicrobial use in cattle. These European Network for Optimization of Veterinary Antimicrobial Therapy (ENOVAT) guidelines provide evidence-based recommendations for antimicrobial and non-steroidal anti-inflammatory drug (NSAID) use in cattle with acute, undifferentiated BRD, balancing animal-level outcomes with antimicrobial stewardship.

**Methods:**

Recommendations were developed using the GRADE approach and Evidence-to-Decision frameworks, following ENOVAT procedures and RIGHT reporting guidance. Evidence came from two prespecified systematic reviews and meta-analyses of randomized controlled trials evaluating individual treatment of clinical BRD: one comparing antimicrobial classes head-to-head and one evaluating NSAIDs as adjunctive treatment or monotherapy. Metaphylaxis and prophylaxis were excluded. Certainty of evidence was assessed with GRADE. Panel judgments incorporated stakeholder-derived thresholds for clinically meaningful differences in short-term therapeutic failure, defined as need for antimicrobial re-treatment, and a merged stewardship perspective based on WHO antimicrobial importance and EMA AMEG categories.

**Results:**

Across antimicrobial comparisons, no class showed a clinically meaningful advantage in reducing short-term re-treatment sufficient to override stewardship considerations. The panel conditionally suggests penicillin G, oxytetracycline, or florfenicol as first-line empirical treatment, guided by local susceptibility data, herd treatment history, product authorization, withdrawal periods, and suspicion of *Mycoplasmopsis bovis* infection. Macrolides are conditionally suggested as second-line treatment, preferably guided by culture and susceptibility testing or documented treatment failure. Fluoroquinolones are recommended against as primary or secondary treatment, except in exceptional, justified circumstances consistent with regulations. Adding NSAIDs to antimicrobials did not meaningfully reduce antimicrobial re-treatment; the panel recommends against NSAID use for this specific purpose. Evidence was insufficient to recommend for or against NSAIDs for welfare-related outcomes or as antimicrobial substitutes.

**Conclusions:**

These guidelines prioritize effective BRD treatment while reducing unnecessary use of higher-priority antimicrobials. Local adoption or adaptation using GRADE-ADOLOPMENT is recommended.

## Plain Language summary

Bovine respiratory disease (BRD) is one of the most common and costly illnesses in cattle. It is usually treated with antibiotics, and many calves also receive painkillers such as non-steroidal anti-inflammatory drugs (NSAIDs). These ENOVAT guidelines help vets and farmers choose treatments that work for animals while protecting antibiotics for the future.

We recommend:

• First choice antibiotics: Penicillin G, oxytetracycline, or florfenicol are reasonable first options for calves with new, undifferentiated BRD. The best choice depends on local experience and test results, what has worked on the farm before, product availability, and whether *Mycoplasmopsis bovis* is suspected.
• Macrolides: Use mainly as a second-line option after a first treatment has failed or when culture/susceptibility results indicate they are needed.
• Fluoroquinolones: Do not use as first- or second-line treatment. Reserve for cases where other options have failed or are not expected to work and only if regulations allow.
• NSAIDs with antibiotics: Do not add an NSAID just to try to prevent a second antibiotic treatment. Studies show this does not make a meaningful difference.
• NSAIDs for comfort: We make no general recommendation for or against NSAIDs to reduce pain or fever. Vets may use them case by case for animals that appear painful or very febrile, following label directions and withdrawal periods.
• NSAIDs instead of antibiotics: We make no general recommendation for or against NSAID-only treatment. If considered, limit to carefully selected, mildly affected animals that can be closely monitored, and start antibiotics promptly if they do not improve.

Across trials, the need for a second antibiotic treatment was similar between many antibiotic classes. That means vets can place more weight on responsible use and local practicality. These guidelines should be adapted to local rules, resistance patterns, and farm conditions, with early reassessment after treatment to ensure animals recover.

## Executive summary (full recommendation and remarks without rationale).

**Table:** 

**Recommendation 1**
**In cattle with acute undifferentiated bovine respiratory disease, we suggest using *either* penicillin G or oxytetracycline or florfenicol as primary empirical treatment. Choice of agent should be guided by local antimicrobial susceptibility data, herd-level treatment history, and whether *Mycoplasmopsis* (formerly *Mycoplasma*) *bovis* is suspected.**
*Conditional recommendation based on very low certainty evidence.*
Remarks: - Penicillin G: Can be considered as primary treatment in settings where penicillin resistance is low, prior penicillin therapy has been clinically effective, and *M. bovis* is not suspected or detected. - Oxytetracycline: Can be considered as primary treatment in settings where oxytetracycline resistance is low, prior oxytetracycline therapy has been clinically effective, and *M. bovis* is suspected or detected. - Florfenicol: Can be considered as primary treatment in settings where florfenicol resistance is low, prior florfenicol therapy has been clinically effective, and *M. bovis* is suspected or detected.
**Recommendation 2**
**In cattle with acute undifferentiated bovine respiratory disease, we suggest that macrolides are reserved as secondary treatment. Selection of a second-line agent should be guided by culture/antimicrobial susceptibility testing when available or by prior antimicrobial exposure and documented treatment failure.**
*Conditional recommendation based on low certainty evidence.*
**Recommendation 3**
**In cattle with acute undifferentiated bovine respiratory disease, we recommend against the use of fluoroquinolones as primary or secondary treatment.**
*Strong recommendation based on low certainty evidence.*
Remark: Fluoroquinolones use should be restricted to cases where antimicrobial resistance against penicillin, oxytetracycline, florfenicol and macrolides is confirmed by clinical or microbiological evidence.
**Recommendation 4**
**In cattle with acute undifferentiated bovine respiratory disease, we recommend against using non-steroidal anti-inflammatory drugs (NSAIDs) in addition to antimicrobials for the specific purpose of preventing antimicrobial re-treatment.**
*Strong recommendation based on high certainty evidence.*
Remark: - This recommendation addresses only the outcome of antimicrobial re-treatment and does not account for analgesia, fever reduction, recovery of appetite or other welfare concerns.
**Recommendation 5**
**In cattle with acute undifferentiated bovine respiratory disease, we make no recommendation for or against the use of NSAIDs for other outcomes (e.g., analgesia, fever reduction, recovery of appetite and other welfare concerns).**
*The certainty of the effect estimates for these outcomes is very low; any direction of effect is highly uncertain. The panel judged that current evidence is insufficient to identify subgroups most likely to benefit from the use of NSAIDs, but agreed that treatment may be required to reduce apparent animal suffering.*
Remark: - Clinicians may consider NSAIDs in addition to antimicrobial treatment on a case-by-case basis for animals assessed to be in pain, balancing potential benefits and harms and adhering to label directions and withdrawal periods.
**Recommendation 6**
**In cattle with acute undifferentiated bovine respiratory disease, we make no recommendation for or against using non-steroidal anti-inflammatory drugs (NSAIDs) as a substitute for antimicrobials.**
*The balance of benefits and harms is likely to vary substantially between individual animals and different herds, and the certainty of evidence is very low. Evidence is insufficient to define subgroups that would reliably benefit; therefore, an overall recommendation is not possible.*
Remarks: - Any potential role for NSAIDs as a stand-alone option should be limited to carefully selected, mildly affected animals which can be closely monitored, with prompt initiation of antimicrobial treatment (rescue therapy) if they fail to improve. - Animals with moderate to severe disease, systemic illness, rapid clinical progression, or comorbidities should not have antimicrobials withheld. - Decisions should be made on a case-by-case basis, considering animal welfare, national guidelines and regulatory requirements, and withdrawal periods.

**Figure 1.**
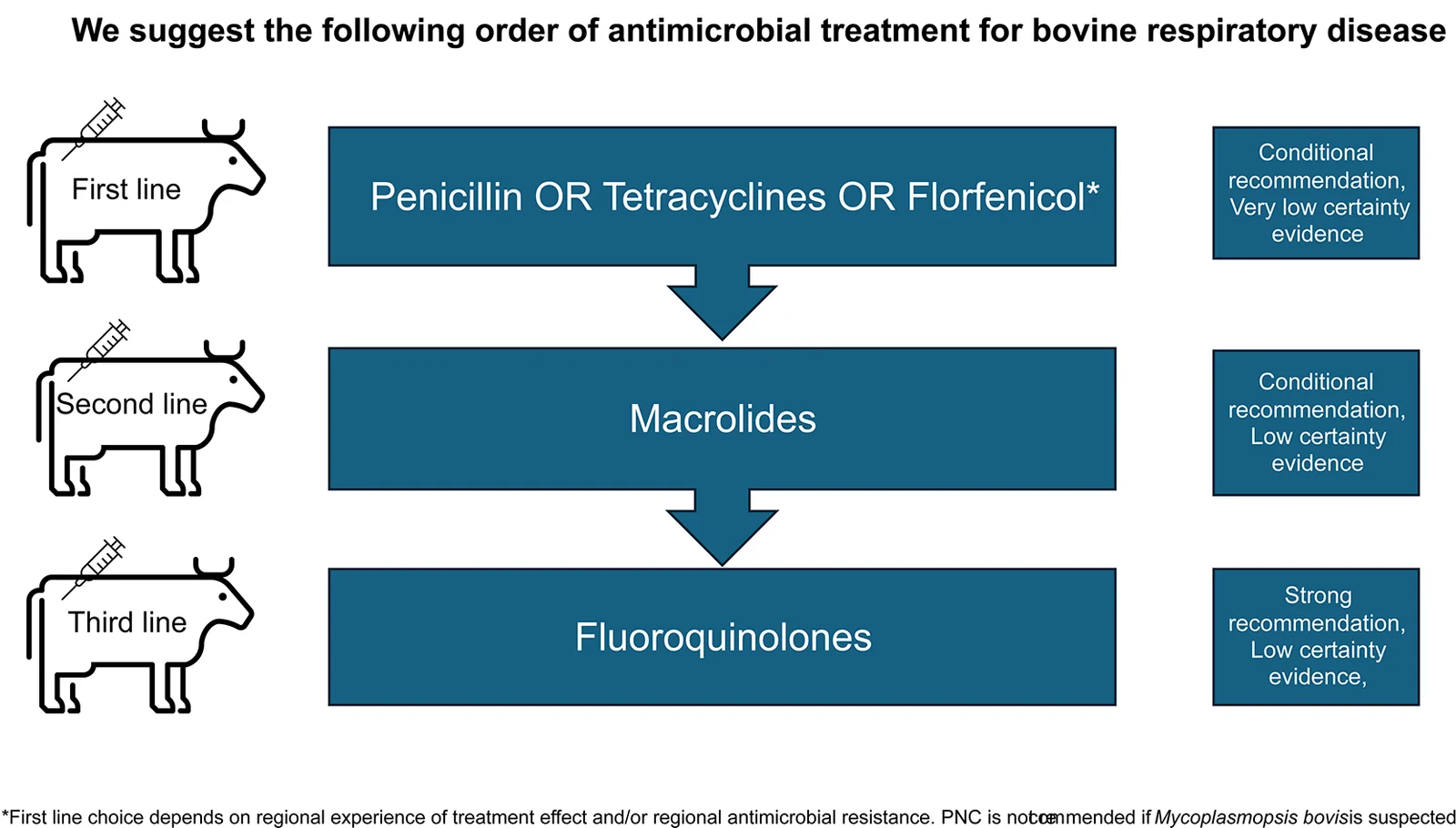
Overview of recommended antimicrobial class placement for empiric treatment of acute, undifferentiated bovine respiratory disease (BRD). First-line classes: penicillins, tetracyclines, phenicols (florfenicol). Second-line: macrolides. Restricted: fluoroquinolones (exceptional, justified circumstances only, consistent with regulations). Recommendation strengths and certainty are shown alongside each class. Abbreviation: BRD = bovine respiratory disease.

Figure 1 provides a visual overview of first-line, second-line, and restricted antimicrobial classes.

## Introduction

Bovine respiratory disease (BRD), an umbrella term for respiratory tract infections in cattle, is a leading cause of morbidity, mortality, production loss, hampered animal welfare and intensive antimicrobial use, worldwide ( O’Donoghue et al., 2025). It arises from interactions among host susceptibility, environmental stressors, and multiple pathogens and is therefore often termed the “bovine respiratory disease complex” ( Bell et al., 2021). Although management factors are integral to pathogenesis, the clinical pneumonia component is frequently attributed to bacterial pathogens or pathobionts, and BRD is consequently treated with antimicrobials in routine field practice. BRD is among the most common indications for antimicrobial use in cattle ( Guardabassi et al., 2018), including agents that are critically important in human medicine ( Pardon et al., 2012; Lhermie et al., 2020). Such use exerts selection pressure for AMR. Multidrug-resistant respiratory bacteria have been reported in some treated populations, although resistance patterns vary and high prevalence AMR does not invariably follow antimicrobial exposure ( Noyes et al., 2015; Catry et al., 2016; Abi Younes et al., 2024). A recent qualitative synthesis of 128 UK ruminant AMU guidelines highlighted persistent “validation, visibility, vagueness and variation” across documents and called for species- and disease-specific guidance, clearer operational definitions, stronger prevention content, and greater alignment across stakeholders ( Best et al., 2023). The present ENOVAT BRD guidance responds to these gaps by providing disease-specific, implementable recommendations intended to reduce unwarranted variation in prescribing while supporting treatment decision-making in BRD.

### Scope and purpose

These guidelines are intended to help veterinary clinicians make evidence-based decisions on the choice of antimicrobial and non-steroidal anti-inflammatory drugs (NSAIDs) for treating cattle with acute, undifferentiated bovine respiratory disease (BRD), balancing animal-level benefits and harms with stewardship considerations. Recommendations are informed by two systematic reviews published by the group ( Scahill et al., 2026a, 2026b). The evidence synthesis included pairwise comparisons among penicillin G, tetracyclines, florfenicol (phenicols), macrolides, and fluoroquinolones for individual treatment of clinical BRD across feedlot, dairy, veal, beef, slaughter, and stocker systems. Trials evaluating metaphylaxis were not included. Companion recommendations address whether to add NSAIDs to antimicrobials or to use NSAIDs as monotherapy in selected cases. While these guidelines are intended for global use, the underlying randomized trials were conducted predominantly in Europe and North America. Direct applicability may vary in regions with different production systems, pathogen/AMR epidemiology, availability and authorization of veterinary medicinal products, and regulatory frameworks. National or regional groups are encouraged to adapt these recommendations to local context using the evidence and reasoning provided. Local adaptation can be undertaken using the ‘GRADE-ADOLOPMENT’ approach (adoption, adaptation, and de novo development), which is a structured method that lets guideline groups adopt existing recommendations and adapt them to local context, or develop new ones while maintaining transparent links to the underlying evidence and Evidence-to-Decision judgments ( Schünemann et al., 2017). This guideline was developed with the support of the European Society of Clinical Microbiology and Infectious Diseases (ESCMID) Study Group for Veterinary Microbiology (ESGVM).

### Interpretation of recommendations and certainty of evidence

Most recommendations in this guideline are supported by very low or low certainty evidence. In GRADE, this typically leads to conditional (rather than strong) recommendations. Even when evidence is sparse, GRADE encourages panels to offer guidance whenever possible; such recommendations will typically be conditional and accompanied by clear remarks. A conditional recommendation should be interpreted as guidance that many stakeholders should be encouraged to follow, but where different choices may be appropriate in some herds or regions after shared decision-making that takes local values, preferences, availability, authorizations, withdrawal periods, costs, and the current international antimicrobial stewardship categorizations of the drugs into account ( World Health Organization, 2024; European Medicines Agency, 2025). Conditional recommendations are also signals that further research is likely to change our confidence in the effect estimates ( Neumann et al., 2026). Conditional recommendations are also signals that further research is likely to change our confidence in the effect estimates ( Neumann et al., 2026).

### National and regional guideline adaptation

GRADE-ADOLOPMENT is a structured, transparent way to take an existing guideline and make it usable in a different setting by either adopting a recommendation as written, adapting it to local realities, or developing a new (de novo) recommendation where gaps exist ( Klugar et al., 2024). As no single international guideline can fit all production systems, regulations, and stakeholder priorities, GRADE-ADOLOPMENT starts from the evidence and the accompanying Evidence-to-Decision (EtD) judgements and asks whether those judgements still hold locally. If they do, a recommendation can be adopted; if local context differs—baseline BRD risk, presence or absence of *M. bovis,* AMR patterns, costs, feasibility, acceptability, equity, or values and preferences—the EtD and the recommendation text or remarks can be adapted, with changes and rationale documented. If no suitable source exists, a de novo recommendation is created, ideally drawing on updated evidence syntheses. To use this guideline with GRADE-ADOLOPMENT, begin with your priority questions, locate the matching recommendation and EtD, add your local data, and record any changes in direction, strength, or remarks. We present absolute effects so users can apply their own thresholds, and we encourage sharing adapted EtDs to support contextualization in other settings.

## Methods

This guideline was produced following the ENOVAT operating procedure ( https://enovat.eu). The Grading of Recommendations, Assessment, Development and Evaluation (GRADE) approach was used to assess the certainty of evidence and to draft recommendations ( Guyatt et al., 2025) and the guidelines are reported according Reporting Items for Practice Guideline in Healthcare (RIGHT) checklist ( Chen et al., 2017).

### Organization, panel composition, planning and coordination

ENOVAT convened a multidisciplinary Drafting Group (DG) to develop these guidelines, drawing on expertise in bovine clinical practice, microbiology, pharmacology, and evidence synthesis/methodology. The group was chaired by LG. The voting panel comprised bovine clinicians (ARW, SB, CLF, MS, BP, RY, LMÅ), a microbiologist (LG), and a pharmacologist (PLT). A methodological oversight committee (KS, LPC, LRJ) harmonized methods across the ENOVAT workstream and a methodology task force (KS, MC, RY, LRJ, LPC) conducted the systematic reviews and meta-analyses, assessed certainty of evidence using GRADE, and prepared Summary of Findings and Evidence-to-Decision materials. Methodology members were non-voting, with the exception of one member who also served on the clinical panel (RY). Panel questions, populations, interventions, and outcomes were refined through an iterative series of virtual meetings and a hybrid workshop. The DG reviewed the systematic review evidence, integrated stakeholder-derived clinical thresholds, and drafted recommendations using GRADE Evidence-to-Decision frameworks, seeking consensus among voting members.

### Guideline funding and the management of competing interests

Consistent with ENOVAT policy, at the beginning of the process all participants were asked to report conflicts of interest (Supplementary File 1). Some participants had received funds for research or consulting from pharmaceutical companies that manufacture and market antimicrobial drugs (BP, SB, ARW), but the degree of involvement was not judged to prohibit their involvement with the process. This project has received funding from the European Union’s Framework Programme for Research & Innovation as part of the COST Action [CA18217, European Network for Optimization of Veterinary Antimicrobial Treatment], as supported by the COST Association (European Cooperation in Science and Technology). No support came from the pharmaceutical industry.

### Selection of questions and outcomes of interest

The antimicrobial classes included in the PICO questions ( Scahill et al., 2026a) were those judged by the DG to represent the classes currently most commonly used for treatment of BRD, excluding ceftiofur in light of the EMA AMEG guidelines ( European Medicines Agency, 2025). Specifically, ceftiofur (a third-generation cephalosporin) is placed in AMEG Category B (Restrict), for which EMA advises use only when Category C (Caution) or D (Prudence) options are not expected to be effective; consequently, ceftiofur head-to-head comparisons were not prioritized for empirical first-line treatment. While a similar argument could be made for the fluoroquinolone class, fluoroquinolones were retained in the assessment because of their perceived importance for treatment of *Mycoplasmopsis* (formerly *Mycoplasma*) *bovis* infections ( Gautier-Bouchardon, 2018). This allowed the guideline panel to consider their clinical effects and to position them appropriately within the EtD from a stewardship perspective. The questions targeted individual treatment of clinically diagnosed, undifferentiated pneumonia (BRD) in cattle across feedlot, dairy, veal, beef, slaughter and stocker systems, and specified direct head-to-head comparisons among penicillins (EMA AMEG Category D), tetracyclines (D), phenicols/florfenicol (C), macrolides (C) and fluoroquinolones (B) across ten pairwise PICOs. Metaphylaxis and prophylaxis were out of scope although prior metaphylaxis was allowed if administered to both the intervention and control group. Pre-specified subgroup analyses considered production system (feedlot vs non-feedlot) and trials including *Mycoplasmopsis*-positive animals, reflecting potential effect modification.

Outcomes were defined *a priori.* Therapeutic failure (failure of clinical disease to resolve within 7 days), relapse, mortality and adverse effects were designated as critical outcomes for decision-making, and liveweight gain as an important (non-critical) outcome. Mortality, relapse definitions beyond re-treatment, adverse effects and liveweight/growth were reported inconsistently and were rarely amenable to pooling results. These limitations informed judgments about indirectness and imprecision in the GRADE assessments.

### Stakeholder involvement and clinical thresholds

We incorporated end-user perspectives to define clinically meaningful effect thresholds and inform Evidence-to-Decision (EtD) judgments. Following GRADE guidance on imprecision ( Zeng et al., 2022), the drafting group developed and piloted a structured questionnaire with panel clinicians, then conducted calibrated telephone interviews with bovine practitioners and cattle producers in multiple countries (Austria, Belgium, Canada, Norway, United States). Stakeholders prioritized outcomes for decision-making and provided three explicit thresholds that classify effects as trivial, small, moderate, or large as well as typical time windows used in practice to judge treatment failure. These thresholds (small effect: 200 animals per 1000; moderate effect 250 animals per 1000, large effect: 300 animals per 1000) were applied to judge imprecision in the systematic reviews and to interpret clinical importance in the EtD frameworks. Stakeholders were recruited by convenience sampling and were predominantly from Europe and North America, which is likely to be a limitation in terms of applicability. The intent was to gather input beyond the perspectives of the panel members, rather than to conduct a fully representative survey and a standalone study.

To our knowledge, this is the first time stakeholder-derived clinical thresholds have been pre-specified and applied in bovine medicine using a GRADE-consistent approach. During panel deliberations we discussed whether the “trivial” and “small” thresholds (e.g., 200 per 1000 animals) might be too high. We decided to retain the original thresholds to maintain transparency. Presenting absolute effects alongside confidence intervals throughout the guideline allows readers and decision makers to apply their own context-specific thresholds (for example, some may regard 100/1000 or even 50/1000 as a “small” effect) and to judge clinical importance accordingly. Detailed methods, instruments, and aggregated threshold results are provided in the antimicrobial systematic review ( Scahill et al., 2026a).

### Generation of recommendations

The recommendations were drafted at a hybrid panel meeting in March 2024. Before the meeting, voting members received: (a) summaries of the ENOVAT systematic review and meta-analysis about antimicrobials with GRADE certainty assessments; (b) results of the veterinarian and farmer threshold surveys; and (c) contextual information relevant to implementation (availability and authorization of veterinary medicinal products, withdrawal times, costs, feasibility across production systems, and stewardship policy frameworks, including WHO/AMEG). Drafting followed the GRADE Evidence-to-Decision (EtD) framework for each question the panel discussed the certainty of the evidence, the balance of desirable and undesirable effects at the animal level, AMR/public health externalities, stakeholder values and preferences, equity, acceptability, feasibility, and resource use ( Alonso-Coello et al., 2016). During the discussions of the stakeholder survey findings, it was noted that many stakeholder queries focused on the role of NSAIDs. In response, the panel agreed to expand the scope to include questions on NSAID adjunct therapy and NSAID monotherapy and commissioned a de novo systematic review.

The panel pre-specified an 80% agreement threshold among voting members to approve a recommendation. Consensus was sought first through discussion; when needed, formal votes were taken during the meeting, with provision for iterative wording thereafter. The final set of recommendations includes one strong recommendation and several conditional recommendations, as well as “no recommendation” statements where the evidence was very uncertain. Draft recommendations, EtD judgments, and manuscript text were reviewed iteratively by the drafting group and voting panel. The final recommendation wording was approved by all voting panel members.

### Stewardship/planetary health perspective in the balance of benefits and harms

The health of humans, animals, and ecosystems is interconnected; guideline decisions that optimize short-term clinical outcomes can still drive long-term harms through AMR and environmental impacts. Recent work within the GRADE community argues that health guidelines should expand beyond a solely individual or public-health lens to explicitly consider planetary health, including AMR, alongside clinical benefits and harms ( Piggott et al., 2026). A GRADE Planetary Health project group have developed methods to integrate these considerations into guideline EtD judgments, while a parallel stewardship initiative is creating a checklist to ensure AMR is systematically addressed during guideline development ( Ibrahim et al., 2026). Consistent with these developments, this guideline pairs individual-animal outcomes with a stewardship perspective in the balance of benefits and harms.

**Figure 2.**
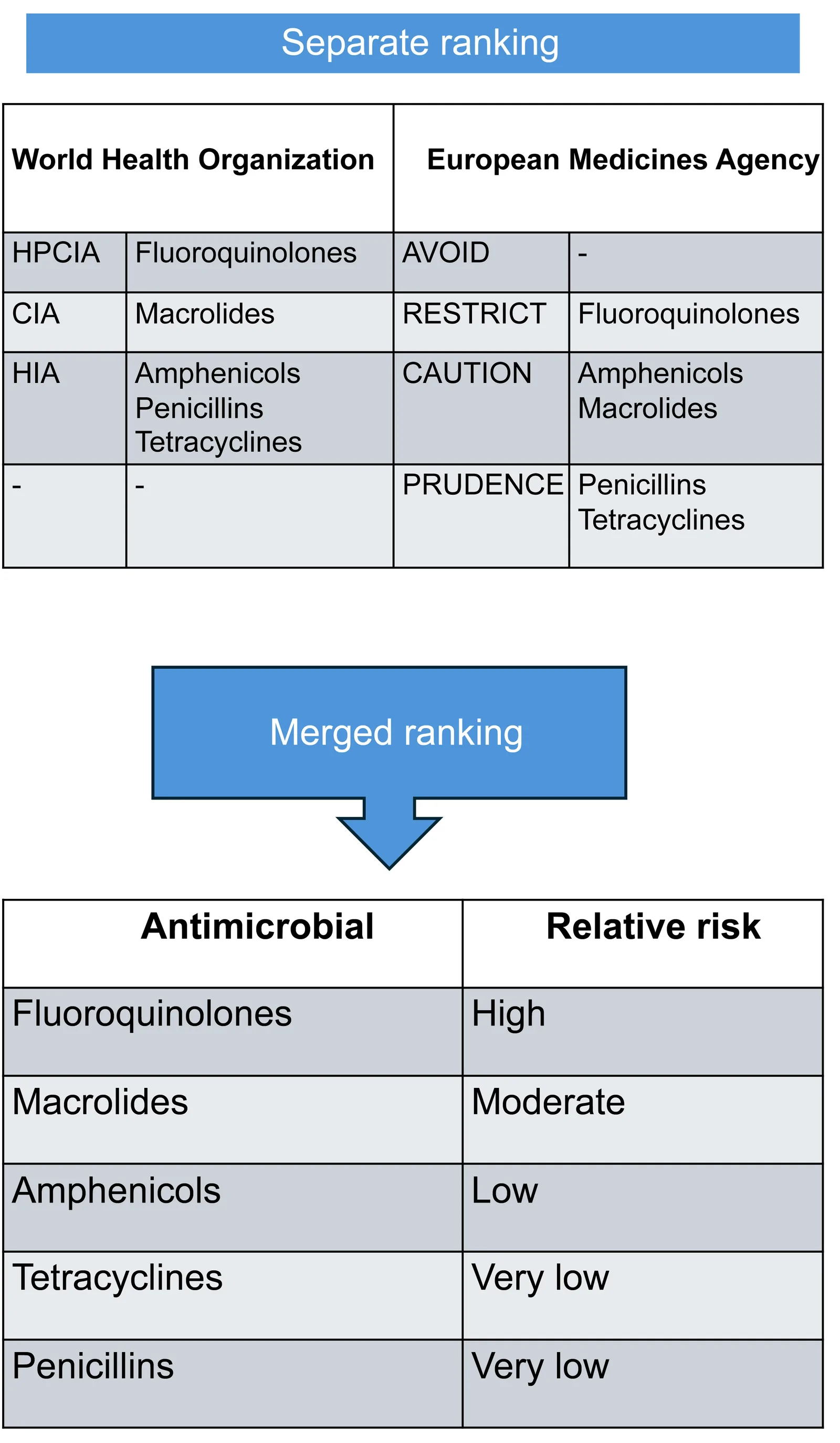
Antimicrobial class prioritization: WHO criticality, EMA/AMEG categories, and the merged stewardship view used in these guidelines. Left: WHO’s list of medically important antimicrobials (human health perspective). Right: EMA/AMEG categorization used in the EU (public health externality plus veterinary need). Below: merged stewardship view applied to EtD judgments in this guideline. Sources: World Health Organization (2024); European Medicines Agency (2025). Abbreviations: AMEG = Antimicrobial Advice ad hoc Expert Group; EMA = European Medicines Agency; WHO = World Health Organization; HP-CIA = Highest Priority Critically Important Antimicrobials; CIA = Critically Important Antimicrobials; HIA = Highly Important Antimicrobials.

There is no single internationally binding prioritization scheme for antimicrobials. The World Health Organization (WHO) categorization ranks classes by their importance for human medicine and the consequences of losing them, taking a human-health perspective ( World Health Organization, 2024). The European Medicines Agency’s (EMA) Antimicrobial Advice ad hoc Expert Group (AMEG) categorization, used in the European Union, weighs both the potential public-health consequences of resistance selection from animal use and veterinary needs. AMEG is embedded in EU legislation and implementation guidance and veterinarians practicing in the EU must adhere to it ( European Medicines Agency, 2025). Outside the EU, regulatory requirements vary widely; in many countries there is no binding legislation specifying antimicrobial class prioritization, and where national or professional guidance exists it should be followed. To reflect global use while remaining compliant for EU readers, we merged these complementary frameworks ( Figure 2). The main difference between these categorizations is that AMEG groups macrolides and florfenicol together (category C), whereas WHO priorities distinguish them, which is relevant to stewardship choices. It is important to note that, even within the EU, individual member states may have stricter stewardship rules and regulations, and the bloc cannot be considered as a homogeneous antimicrobial user.

### Multiple comparison EtD framework

We summarized panel judgments across EtD criteria using the new GRADE EtD framework for multiple comparisons ( Beltrán et al., 2026). For the antimicrobial class question, we pre-specified critical/important outcomes and decision thresholds (for short-term re-treatment) and populated a multi-comparison Summary of Judgments (SoJ) table for the head-to-head pairs evaluated in the SR (PICOs 1, 3, 5–10; Table 1). For two pairs (PNC vs MAC; PNC vs QUI) no direct randomized treatment trials were found and these comparisons were not included. Domains were judged by the panel using SR estimates (desirable/undesirable effects, net effect, certainty of effects) and, where relevant, a merged WHO–AMEG stewardship perspective for planetary health/AMR. Context-dependent criteria (resources/costs, cost-effectiveness, acceptability, feasibility, and planetary-health implementation issues) were recorded as global baseline EtD judgments from the panel; we did not conduct separate systematic reviews for these domains, and users are encouraged to adapt them locally during ADOLOPMENT. For the NSAID comparisons (adjunct vs none; monotherapy vs antimicrobial), the single-comparison EtD tables likewise include panel baseline judgments (not SRs) for these context domains ( Table 2).

**Table 1.** This table presents GRADE Evidence-to-Decision (EtD) Summary of Judgments for pairwise antimicrobial class comparisons included in the antimicrobial systematic review (SR), ordered by PICO: PICO 1 (PNC vs TET), PICO 3 (PNC vs FLO), PICO 5 (TET vs MAC), PICO 6 (TET vs FLO), PICO 7 (TET vs QUI), PICO 8 (FLO vs MAC), PICO 9 (MAC vs QUI), and PICO 10 (FLO vs QUI). PICO 2 (PNC vs MAC) and PICO 4 (PNC vs QUI) are not shown because no direct randomized head-to-head treatment trials were identified. Judgments for “Desirable/Undesirable effects” and the resulting “Net effect” are based on absolute effects against stakeholder-derived decision thresholds for short-term therapeutic failure (re-treatment) (trivial <200 per 1000; small 200–250; moderate 250–300; large >300). “Certainty of evidence of effects” reflects the SR’s GRADE ratings across critical outcomes for each comparison. “Balance of effects” integrates net effects and certainty to indicate whether either option is favored. “Resources required,” “Acceptability,” and “Feasibility” reflect global baseline judgments and may vary by jurisdiction and production system; users should adapt these domains locally (ADOLOPMENT). The “Planetary health/Risk of antimicrobial resistance” row indicates the relative AMR externality risk for each class (intervention vs comparator) using a merged WHO–AMEG stewardship perspective: penicillins and tetracyclines = very low; phenicols (florfenicol) = low; macrolides = moderate; fluoroquinolones = high. Re-treatment is used as a surrogate for short-term treatment failure; long-horizon outcomes (mortality, distant relapse, performance) were inconsistently attributable to the index drug.

Criterion	Signaling questions	PNC vs TET (PICO 1)	PNC vs FLO (PICO 3)	TET vs MAC (PICO 5)	TET vs FLO (PICO 6)	TET vs QUI (PICO 7)	FLO vs MAC (PICO 8)	MAC vs QUI (PICO 9)	FLO vs QUI (PICO 10)
Problem	Is the problem a priority?	Yes	Yes	Yes	Yes	Yes	Yes	Yes	Yes
Desirable health and well-being effects	How substantial are the desirable effects for each comparison?	Trivial to no effect	Trivial to no effect	Trivial to no effect	Trivial to no effect	Trivial to small	Trivial to no effect	Trivial to no effect	Trivial to no effect
Undesirable health and well-being effects	How substantial are the undesirable effects for each comparison?	Trivial to no effect	Trivial to no effect	Trivial to no effect	Trivial to no effect	Trivial to small	Trivial to no effect	Trivial to no effect	Trivial to no effect
Net effect	How substantial is the magnitude of desirable vs undesirable effects (each comparison)?	Null or trivial net effect	Null or trivial net effect	Null or trivial net effect	Null or trivial net effect	Null or trivial net effect	Null or trivial net effect	Null or trivial net effect	Null or trivial net effect
Certainty of evidence of effects	Overall certainty across critical outcomes for each comparison	Very low	Low	Very low	Low	Very low	Moderate	Moderate	Low
Certainty of evidence of values	Is there important uncertainty about how much outcomes are valued?	Probably no important uncertainty	Probably no important uncertainty	Probably no important uncertainty	Probably no important uncertainty	Probably no important uncertainty	Probably no important uncertainty	Probably no important uncertainty	Probably no important uncertainty
Balance of effects	Does the balance favor intervention or comparator?	Does not favor either	Does not favor either	Does not favor either	Does not favor either	Does not favor either	Does not favor either	Does not favor either	Does not favor either
Resources required	How large are resource requirements (costs)?	Negligible costs and savings	Negligible costs and savings	Negligible costs and savings	Negligible costs and savings	Negligible costs and savings	Negligible costs and savings	Negligible costs and savings	Negligible costs and savings
Certainty of evidence of resources	Certainty in resource estimates	Very low	Very low	Very low	Very low	Very low	Very low	Very low	Very low
Planetary health/Risk of antimicrobial resistance	Risk group in intervention and comparator	Very low vs very low	Very low vs low	Very low vs moderate	Very low vs low	Very low vs high	Low vs moderate	Moderate vs high	Low vs high
Acceptability	Acceptability to key interest holders	Varies	Varies	Varies	Varies	Varies	Varies	Varies	Varies
Feasibility	Feasibility to implement	Probably yes (but lack of long-acting PNC makes it less feasible than TET)	Probably yes	Probably yes	Probably yes	Probably yes	Probably yes	Probably yes	Probably yes

Abbreviations: PNC = penicillins; TET = tetracyclines; FLO = phenicols (florfenicol); MAC = macrolides; QUI = fluoroquinolones.

Note: While feasibility is “Probably yes” across most comparisons, lack of long-acting penicillin formulations may reduce practicality relative to long-acting tetracycline/phenicol options in some settings.

**Table 2.** Summary of Judgments table presents panel judgments for two NSAID comparisons: adjunct therapy (NSAID + antimicrobial [ATB] vs ATB alone) and monotherapy (NSAID vs ATB). Judgments for “Desirable/Undesirable effects” and the resulting “Net effect” are based on absolute effects for short-term therapeutic failure (re-treatment), assessed against stakeholder-derived decision thresholds (trivial <200 per 1000; small 200–250; moderate 250–300; large >300). Certainty of evidence reflects the NSAID SR: high certainty of no clinically meaningful benefit for adjunct use on re-treatment; very low certainty (extremely serious imprecision) for NSAID monotherapy. Context-dependent criteria (resources/cost-effectiveness, acceptability, feasibility, planetary-health implementation issues) are global baseline panel judgments—no separate systematic reviews were conducted for these domains—and should be adapted locally during ADOLOPMENT. NSAIDs have no AMR externality; however, avoidable use adds environmental footprint (manufacturing, packaging, distribution, waste). Welfare outcomes (analgesia/antipyresis/comfort) were heterogeneously reported and not poolable; these are addressed narratively in Recommendation 5.

Criterion	Signaling questions	ATB + NSAID vs ATB (adjunct to prevent re-treatment)	NSAID vs ATB (monotherapy)
Problem	Is the problem a priority?	Yes	Yes
Desirable health and well-being effects	How substantial are the desirable effects for each comparison?	Trivial to no effect (re-treatment)	Trivial to no effect (very uncertain)
Undesirable health and well-being effects	How substantial are the undesirable effects for each comparison?	Trivial to no effect (AEs sparsely reported)	Trivial to small (risk of undertreatment; very uncertain)
Net effect	How substantial is the magnitude of desirable vs undesirable effects (each comparison)?	Null or trivial net effect (for re-treatment)	Uncertain (very low certainty)
Certainty of evidence of effects	Overall certainty across critical outcomes for each comparison	High (for re-treatment) and very low (for other outcomes)	Very low
Certainty of evidence of values	Is there important uncertainty about how much outcomes are valued?	Probably no important uncertainty (for re-treatment)	Possibly important uncertainty
Balance of effects	Does the balance favor intervention or comparator?	Does not favor either (for re-treatment)	Uncertain/Does not favor either
Resources required	How large are resource requirements (costs)?	Negligible costs and savings (global baseline)	NSAID monotherapy is likely more resource intense due to extended monitoring
Certainty of evidence of resources	Certainty in resource estimates	Very low	Very low
Planetary health/Risk of antimicrobial resistance	Risk group in intervention and comparator	Same AMR externality (ATB in both) + added NSAID footprint	None (NSAID) vs class-dependent (antimicrobial)
Acceptability	Acceptability to key interest holders	Varies	Varies
Feasibility	Feasibility to implement	Probably yes	Varies

Abbreviations: ATB = antimicrobial; NSAID = non-steroidal anti-inflammatory drug; BRD = bovine respiratory disease.

### Consultation phase

A one-month stakeholder consultation will be conducted independently of the Open Research Europe (ORE) public commenting and peer review processes. Invited stakeholders will be contacted by email and provided with the Version 1 manuscript and instructions for submitting written comments directly to the authors. Stakeholder input will not automatically change the recommendations; rather, it will be reviewed by the guideline panel and used to contextualize and transparently document areas of agreement and disagreement, for example regarding acceptability, feasibility, costs, implementation barriers, and local applicability.

A summary of the consultation process and the main themes raised will be added to Version 2 of the manuscript. Comments and author responses will be made available as extended data. Examples of invited groups include national and regional veterinary bodies, buiatrics associations, producer organizations, veterinary colleges and federations, and reference agencies; the full invite list is provided in Supplementary File 2.

## Results

### Recommendation 1

**In cattle with acute undifferentiated bovine respiratory disease, we suggest using *either* penicillin G *or* oxytetracycline *or* florfenicol as primary empirical treatment. Choice of agent should be guided by local antimicrobial susceptibility data, herd-level treatment history, and whether *M. bovis* is suspected.**

*Conditional recommendation based on very low certainty evidence (100% agreement).*

Remarks:

- Penicillin G: Can be considered as primary treatment in settings where penicillin resistance is low and/or prior penicillin therapy has been clinically effective, and *M. bovis* is not suspected or detected.
- Oxytetracycline: Can be considered as primary treatment in settings where oxytetracycline resistance is low and/or prior oxytetracycline therapy has been clinically effective, and *M. bovis* is suspected or detected.
- Florfenicol: Can be considered as primary treatment in settings where florfenicol resistance is low and/or prior florfenicol therapy has been clinically effective, and *M. bovis* is suspected or detected.

### Rationale for Recommendation 1

Summary of the evidence, benefits, and harms.

The ENOVAT antimicrobial systematic review and meta-analysis compared five commonly used antimicrobial classes in head-to-head randomized trials for individual treatment of clinical BRD. As previously described in the systematic review ( Scahill et al., 2026a), across the relevant pairwise comparisons informing first-line choices, no class showed a clinically meaningful advantage in reducing short-term treatment failure (defined as need for re-treatment), based on stakeholder-derived clinical thresholds:

• Penicillin G vs tetracyclines: RR 0.97 (95% CI 0.76–1.25), 8 fewer treatment failures per 1000 animals (from 65 fewer to 67 more); very low certainty.
• Penicillin G vs florfenicol: RR 0.66 (0.16–2.75), 38 fewer per 1000 (from 93 fewer to 194 more); low certainty.
• Tetracyclines vs florfenicol: RR 0.96 (95% CI 0.70–1.30), 7 fewer per 1000 animals (from 53 fewer to 53 more); low certainty.

When comparing these first-line candidates against higher-priority classes, estimates likewise did not demonstrate a clinically relevant advantage for macrolides or fluoroquinolones:

• Florfenicol vs macrolides: RR 1.10 (0.94–1.28), 27 more per 1000 (from 16 fewer to 75 more); moderate certainty.
• Tetracyclines vs macrolides: RR 1.45 (0.87–2.41), 132 more per 1000 (from 38 fewer to 415 more); very low certainty.
• Florfenicol vs fluoroquinolones: RR 0.58 (0.32–1.04), 102 fewer per 1000 (from 165 fewer to 10 more); low certainty.
• Tetracyclines vs fluoroquinolones: RR 1.45 (1.15–1.84), 179 more per 1000 (from 60 more to 343 more); very low certainty.

Subgroup analyses (feedlot vs non-feedlot; trials with *Mycoplasmopsis*-positive animals) did not change conclusions. Mortality, relapse beyond the first re-treatment, adverse effects, and growth were inconsistently reported over prolonged follow-up with frequent cross-over and rescue therapy; these outcomes were not reliably attributable to the initial randomized drug and contributed to indirectness and, in some cases, imprecision. Direct animal-level adverse events were uncommon and rarely influenced the balance of benefits and harms. Consequently, when short-term efficacy appeared similar, the panel incorporated explicit stewardship considerations (WHO–AMEG merged perspective; Figure 2) alongside local implementation factors. Table 1 presents the GRADE EtD Summary of Judgments for the antimicrobial class head-to-heads included in the SR (PICOs 1, 3, 5–10).

***Macrolide-specific clarification***

Our decision not to include macrolides as first-line treatment is not driven by the very low-certainty tetracycline vs macrolide comparison in which tilmicosin was the sole macrolide (PICO 5). Rather, it rests on PICO 8 (florfenicol vs macrolides), which pooled trials including both tulathromycin and tilmicosin (and one gamithromycin trial) and showed no clinically meaningful advantage of macrolides over florfenicol: RR 1.10 (95% CI 0.94–1.28), corresponding to 27 more re-treatments per 1000 animals (from 16 fewer to 75 more); moderate certainty. To address the concern that macrolides should not be treated as a homogeneous group, we ran sensitivity analyses by macrolide ( Table 3). Forest plots are presented in Supplementary File 3. Although the recommendation was primarily based on the absence of a clinically meaningful efficacy advantage of macrolides, their higher priority ranking in the WHO and AMEG classifications ( Figure 2) was also considered during the panel’s deliberations.

**Table 3.** Agent-specific sensitivity within PICO 8 (florfenicol vs macrolides). Absolute effects and risk ratios for short-term re-treatment comparing (A) florfenicol vs tilmicosin and (B) florfenicol vs tulathromycin against prespecified clinical thresholds. Outcome: first re-treatment within each trial’s observation window.

Comparison	Absolute effect	RR (95% CI)	p-value
FLO vs TIL	FLO treatment resulted in 41 fewer treatment failures per 1000 animals (from 102 fewer to 38 more) in comparison to TIL	0.87 (0.68–1.12)	0.14
FLO vs TUL	FLO treatment resulted in 51 more treatment failures per 1000 animals (from 21 fewer to 138 more) in comparison to TUL	1.19 (0.92–1.52)	0.14

Abbreviations: FLO = florfenicol; TIL = tilmicosin; TUL = tulathromycin.

Tulathromycin performed marginally better than tilmicosin when compared to florfenicol, but neither subset demonstrated a clinically relevant advantage for either substance over florfenicol against our prespecified thresholds, and confidence intervals encompassed trivial effects. Taken together with stewardship considerations, this supports positioning macrolides as a second-line class rather than first-line in undifferentiated clinical BRD.

### Applicability, feasibility, and acceptability

Product authorization and availability, withdrawal periods, local AMR patterns, prior herd-level response, costs, and handling/logistics vary widely and should guide first-line choice. The evidence base, stakeholder input, and panel composition were predominantly European and North American; non-English language studies were not included. Direct applicability may therefore be constrained in other regions with different production systems, epidemiology, and product landscapes. Routine first-line use of penicillin G for BRD is currently largely confined to Scandinavian countries, where panelists reported lower perceived resistance in target pathogens and satisfactory field response. Outside this region, recent clinical experience with penicillin G for BRD is limited and some clinicians expressed concerns regarding the practicality of administering penicillin G on a daily basis. These factors reduce acceptability and practicality in many settings, and they contributed to including florfenicol alongside penicillin G and oxytetracycline as acceptable first-line options in this guideline.

### Other decision criteria and considerations

Planetary health/AMR: Where short-term efficacy is comparable, a stewardship tilt favors lower externality-risk classes. (NB: Externality risk refers to the expected harm that the use of a particular antimicrobial imposes on parties other than the treated patient or animal). In our merged overview, penicillins and tetracyclines carry very low externality risk and phenicols low risk; macrolides are higher and fluoroquinolones highest. Consistent with the recommendation, florfenicol was retained as a first-line option alongside penicillin G and oxytetracycline. This reflects both stewardship balancing and practical realities: in many European settings tetracycline resistance is perceived to be common in respiratory pathogens (particularly when *M.bovis* is suspected), reducing acceptance of a narrow first-line set limited to penicillin/tetracycline. Emerging laboratory surveillance and systematic-review evidence (manuscript in preparation) also suggest high *in vitro* resistance to tetracyclines in some contexts; while indirect, this informed acceptability supports inclusion of florfenicol as a reasonable first-line alternative within a stewardship-conscious framework.

### Certainty in the net effect

Certainty for individual comparisons ranged from very low to moderate in the systematic review, the panel judged overall certainty in the net effect for this recommendation to be very low. This reflects (a) indirectness due to reliance on a surrogate (re-treatment) and limited attribution of long-horizon outcomes; (b) risk of bias and imprecision in several comparisons; and (c) generalizability concerns for international use, given that trials, stakeholder thresholds, and panel perspectives were largely European/North American and non-English language studies were excluded. These factors support a conditional recommendation and emphasize local adaptation and early clinical reassessment.

### Conclusion

Given the absence of clinically meaningful differences in short-term re-treatment across compared classes, and considering stewardship priorities, practical acceptability in the field (including concerns about tetracycline resistance in some settings), and implementation realities, the panel suggests penicillin G, oxytetracycline, or florfenicol as appropriate first-line empirical choices for acute, undifferentiated BRD. Decision-making between these three antimicrobials should be guided by local susceptibility data, herd-level treatment history, authorized products and withdrawal periods, and whether *Mycoplasmopsis bovis* is suspected. We view this document as an example guideline: national or regional groups should adapt recommendations to their local context using the GRADE-ADOLOPMENT approach (adopt/adapt/de novo) and engage local end-users, particularly farmers and veterinarians to ensure feasibility and acceptance.

### Recommendation 2

**In cattle with acute undifferentiated bovine respiratory disease, we suggest that macrolides are reserved as a secondary treatment. The selection of a second-line agent should be guided by culture/antimicrobial susceptibility testing when available or by prior antimicrobial exposure and documented treatment failure.**

*Conditional recommendation based on low certainty evidence. (100% agreement).*

### Rationale for Recommendation 2

This suggestion to reserve macrolides as second-line is anchored in the florfenicol versus macrolide comparison (PICO 8), which pools head-to-head randomized trials across production systems and includes both tulathromycin and tilmicosin (plus one gamithromycin trial). In the primary analysis, macrolides as a whole did not demonstrate a clinically meaningful advantage over florfenicol for short-term re-treatment. Florfenicol treatment resulted in 27 more treatment failures per 1000 animals (from 16 fewer to 75 more) in comparison to macrolides; moderate certainty evidence.

### Within class macrolide analysis

The panel does not recommend a specific macrolide over another as this extends beyond the scope of this guideline, but addresses the concerns about treating macrolides as a homogeneous class. We complemented the class-level florfenicol vs macrolide findings with two targeted explorations. First, we examined agent-specific subsets within PICO 8 to see whether the pooled class effect was driven by a particular macrolide. Second, because PICO 8 does not directly compare macrolides with each other, we conducted a targeted rapid review of peer-reviewed head-to-head trials comparing tulathromycin with tilmicosin.

*PICO 8 sensitivity analysis*

Within PICO 8 (florfenicol vs macrolide), we split the pooled macrolide group by agent (tulathromycin vs florfenicol and tilmicosin vs florfenicol) to determine whether the class-level result masked clinically relevant differences between commonly used tulathromycin and tilmicosin ( Table 3). Although the directions differ slightly (favoring florfenicol over tilmicosin; favoring tulathromycin over florfenicol), neither analysis shows a clinically meaningful advantage over florfenicol against our predefined thresholds. These findings reinforce that macrolides should not be prioritized over florfenicol for first-line empirical treatment of undifferentiated clinical BRD. Forest plots are provided in Supplementary File 3.

*Rapid review of pairwise analysis (tulathromycin vs tilmicosin)*

To further address within-class concerns (see peer review comments in Scahill et al., 2026a), we conducted a *post hoc* rapid review of peer-reviewed therapeutic RCTs directly comparing tulathromycin with tilmicosin. Three trials ( Kilgore et al., 2005; Nutsch et al., 2005; Skogerboe et al., 2005) contributed to the primary meta-analysis, enrolling 735 animals in the tulathromycin arm and 729 in the tilmicosin arm. Results are summarized in Table 4 and show effects within the prespecified trivial threshold for clinical importance, i.e., no clinically meaningful advantage for tulathromycin over tilmicosin on first re-treatment. A sensitivity analysis adding a peer-reviewed macrolide context trial (gamithromycin vs tilmicosin; Van Donkersgoed and Hendrick, 2013) did not change this interpretation. Full methods and study details are provided in Supplementary File 3. A second sensitivity analysis added tulathromycin–tilmicosin event counts abstracted second-hand from Wellman and O’Connor (2007), which summarized manufacturer technical reports unavailable to us, alongside the peer-reviewed macrolide context trial ( Van Donkersgoed and Hendrick, 2013). Including these reports increased totals to tulathromycin/gamithromycin n = 1,663 (305 events) versus tilmicosin n = 1,657 (537 events). The pooled estimate remained within our prespecified trivial effect range (see Table 4).

**Table 4.** Pairwise macrolide comparisons (tulathromycin/gamithromycin vs tilmicosin). Primary analysis: peer-reviewed RCTs only. Sensitivity analysis: includes tulathromycin–tilmicosin counts abstracted second-hand from Wellman and O’Connor (2007), which summarizes manufacturer technical reports unavailable to us. Outcome: first re-treatment within each trial’s observation window.

Comparison	Absolute effect	RR (95% CI)	p-value
TUL vs TIL (only peer-reviewed)	TUL treatment resulted in 64 fewer treatment failures per 1000 animals (from 125 fewer to 29 more) in comparison to TIL	0.76 (0.53–1.11)	0.15
TUL/GAM vs TIL (only peer-reviewed)	TUL/GAM treatment resulted in 40 fewer treatment failures per 1000 animals (from 85 fewer to 21 more) in comparison to TIL	0.81 (0.6–1.10)	0.18
TUL/GAM vs TIL including technical reports	TUL/GAM treatment resulted in 97 fewer treatment failures per 1000 animals (from 169 fewer to 6 more) in comparison to TIL	0.70 (0.48–1.02)	0.07

Abbreviations: TUL = tulathromycin; GAM = gamithromycin; TIL = tilmicosin.

### Other decision criteria and considerations

• Indirectness of “second-line” positioning: PICO 8 trials evaluated first-line use; our recommendation concerns order of use (reserving macrolides for second line). We therefore downgraded one additional level for indirectness when moving from the moderate-certainty first-line estimate to a second-line placement resulting in low certainty evidence.
• Stewardship/AMR: In the WHO–AMEG merged stewardship perspective used throughout, macrolides carry a higher AMR externality risk than penicillins, tetracyclines, and phenicols. Where short-term efficacy is comparable, stewardship considerations weigh against routine first-line macrolide use.
• Implementation: When macrolides are used second line, agent choice should be guided by culture/susceptibility testing where feasible, or by prior antimicrobial exposure and documented treatment failure, together with local authorization, withdrawal periods, availability, costs, and feasibility on farm. Consistent with this class-level placement, our agent-specific exploration did not identify a clinically meaningful within-class advantage, so choice between macrolides should be based on local susceptibility, prior exposure, and product authorizations rather than presumed efficacy differences.

### Certainty in the net effect

Although the first-line florfenicol versus macrolide comparison (with all macrolides pooled) was of moderate certainty, the question here is second-line use. We therefore downgraded one more level for indirectness (it was previously downgraded one level for the re-treatment surrogate outcome), resulting in low certainty for this recommendation. This judgment also reflects the broader limitations summarized in Recommendation 1 (surrogate primary outcome; inconsistent long-horizon outcomes; risk of bias and imprecision in some trials; and generalizability concerns given the Europe/North America evidence base and English-language restriction). We also considered two within-class explorations: (a) agent-specific PICO 8 sensitivity analyses (florfenicol vs tilmicosin; florfenicol vs tulathromycin), which stayed within the prespecified trivial effect range; and (b) a post hoc rapid review of peer-reviewed tulathromycin vs tilmicosin trials, which did not show a clinically meaningful within-class advantage. These findings supported the class-level, second-line placement but did not change the overall low certainty in the net effect.

### Conclusion

Given the absence of a clinically meaningful advantage for macrolides over florfenicol on short-term re-treatment in randomized trials, together with stewardship considerations and the indirectness of applying first-line evidence to second-line positioning, the panel suggests reserving macrolides as secondary treatment in acute, undifferentiated BRD. Selection of a second-line agent should be guided by culture/susceptibility testing when available, or by prior antimicrobial exposure with documented treatment failure, and by authorization, withdrawal periods, feasibility, and farm context. Our within-class analyses did not identify a clinically meaningful advantage of tulathromycin over tilmicosin (nor of macrolides over florfenicol), reinforcing this class-level placement; choice between macrolides should therefore reflect local data and practical constraints.

As this is a conditional recommendation, exceptions are expected. In high-risk herds or outbreaks where florfenicol efficacy appears reduced, where local surveillance or farm-level data suggest florfenicol resistance, or where authorization/availability constraints preclude phenicol use, macrolides may reasonably be used earlier following shared decision-making and early clinical reassessment.

### Recommendation 3

**In cattle with acute undifferentiated bovine respiratory disease, we recommend against the use of fluoroquinolones as primary or secondary treatment.**

*Strong recommendation based on low certainty evidence (100% agreement).*

Remark

- Fluoroquinolones use should be limited to use when AMR against penicillin, oxytetracycline, florfenicol and macrolides is documented by clinical or microbiological evidence.

### Rationale for Recommendation 3

Summary of the evidence, benefits, and harms.

This recommendation draws on two direct, head-to-head comparisons that address whether fluoroquinolones offer a short-term efficacy advantage for individual treatment of clinical BRD:

• PICO 9 (macrolides vs fluoroquinolones): macrolides did not perform worse than fluoroquinolones; if anything, they tended towards fewer re-treatments. RR 0.79 (95% CI 0.54–1.16), corresponding to 48 fewer re-treatments per 1000 animals (from 106 fewer to 37 more); moderate certainty. The absolute effect lies within the prespecified trivial range for clinical importance.
• PICO 10 (florfenicol vs fluoroquinolones): florfenicol did not perform worse than fluoroquinolones and tended towards fewer re-treatments. RR 0.58 (95% CI 0.32–1.04), corresponding to 102 fewer per 1000 (from 165 fewer to 10 more); low certainty. The absolute effect remains within the trivial range.
• PICO 7 (tetracyclines vs fluoroquinolones): Tetracyclines RR 1.45 (95% CI 1.15–1.84) with an absolute difference of 179 more per treatment failures per 1000 animals for tetracyclines in comparison to fluoroquinolones; this is statistically significant (p < 0.05) but still below our “small” effect threshold (≥200 per 1000) and based on very low certainty, and therefore does not offset the lack of advantage for fluoroquinolones over macrolides or florfenicol in the primary comparisons.

### Other decision criteria and considerations

• Stewardship/AMR and planetary health: Fluoroquinolones are WHO Highest Priority Critically Important Antimicrobials for human medicine and are placed in EMA AMEG Category B (Restrict). In our merged WHO–AMEG stewardship perspective, they carry the highest AMR externality risk among the classes assessed. Where short-term efficacy is comparable (i.e., no clinically meaningful advantage on re-treatment), stewardship strongly weighs against routine first- or second-line use to preserve effectiveness for human and veterinary medicine and to minimize AMR externalities.
• Policy and regulations: In the EU, AMEG Category B agents are subject to legal restrictions; veterinarians must prioritize Category D/C options, where effective. Outside the EU, requirements vary; where no binding prioritization exists, the same stewardship logic applies.
• Implementation: If fluoroquinolones are considered, this should be exceptional and justified (e.g., culture/susceptibility demonstrating resistance to, or documented failure of, lower-risk options such as penicillins, tetracyclines, phenicols, and macrolides), with early clinical reassessment, adherence to product authorization and withdrawal periods, and clear documentation of rationale consistent with national rules.

### Certainty in the net effect

The key comparisons for this question provide moderate certainty (macrolides vs fluoroquinolones) and low certainty (florfenicol vs fluoroquinolones) that fluoroquinolones do not confer a clinically meaningful reduction in short-term re-treatment. Considering these together, the panel judged certainty in the net effect sufficient to support a strong recommendation against routine use because:

• the direct head-to-head evidence does not show a clinically important efficacy advantage for fluoroquinolones over macrolides or florfenicol; and
• the AMR/public- and planetary-health harms and regulatory constraints associated with fluoroquinolones are substantial.

This aligns with GRADE situations in which a strong recommendation is appropriate despite less-than-high certainty in all effect estimates: when benefits are similar but one option entails clearly greater harms or policy costs (here: AMR externalities and legal constraints), the net effect favors avoiding the higher-risk option.

### Conclusion

Given the absence of a clinically meaningful short-term efficacy advantage for fluoroquinolones over macrolides (moderate certainty) or florfenicol (low certainty), together with their high AMR externality risk (WHO Highest Priority; AMEG Category B Restrict) ( Figure 2) and, in the EU, legal constraints, the panel strongly recommends against using fluoroquinolones as primary or secondary empirical treatment for acute, undifferentiated BRD. In exceptional circumstances (e.g., culture-confirmed resistance to, or documented failure of, lower-risk options; life-threatening disease where no alternatives are available and permitted), earlier use may be justified under national regulations, with early reassessment and clear documentation of rationale.

### Recommendation 4

**In cattle with acute undifferentiated bovine respiratory disease, we recommend against using non-steroidal anti-inflammatory drugs (NSAIDs) in addition to antimicrobials for the specific purpose of preventing antimicrobial re-treatment.**

*Strong recommendation based on high-certainty evidence (100% agreement).*

Remarks:

- This recommendation addresses only the outcome of antimicrobial re‐treatment and does not address analgesia, fever reduction or recovery of appetite.

### Rationale for Recommendation 4

Summary of the evidence, benefits, and harms.

This recommendation addresses a focused question: does adding an NSAID to antimicrobial therapy reduce the need for antimicrobial re-treatment in acute, undifferentiated BRD? Across 17 randomized trials (22 comparisons; ~4,900 animals), adding an NSAID did not reduce re-treatment versus antimicrobials alone: RR 0.94 (95% CI 0.84–1.05), absolute difference 12 fewer per 1000 (from 31 fewer to 10 more); I2 = 0%. Findings were robust in sensitivity analyses restricting follow-up to ≤14 days (RR 0.85, 95% CI 0.70–1.03) and ≤ 10 days (RR 0.84, 95% CI 0.69–1.03), in analyses limited to low risk-of-bias trials (RR 0.99, 95% CI 0.86–1.14), and in subgroup analyses (same antimicrobial in both arms vs different; flunixin-only vs other NSAIDs), all of which remained within the prespecified trivial effect range. Funnel plot inspection did not suggest publication bias. The GRADE EtD Summary of Judgments ( Table 2) shows no clinically meaningful reduction in re-treatment with NSAID adjuncts (high certainty), a null/trivial net effect, and a balance that does not favor adding NSAIDs for this purpose.

Typically, guideline panels weigh all critical outcomes jointly and not in isolation. However, in many field settings NSAIDs are given with the expectation that they prevent antimicrobial re-treatment; the panel therefore issued this focused recommendation to correct that specific misconception. On this outcome, the evidence consistently shows no clinically meaningful benefit. The panel recognizes that NSAIDs may be used for animal-welfare indications (analgesia, antipyresis, improved comfort/appetite), which are outside the scope of this recommendation and addressed separately (Recommendation 5).

### Other decision criteria and considerations

• Harms, burden, resources, and planetary health: Trial reports of NSAID-related adverse events were sparse, and NSAIDs do not carry AMR externality concerns. However, unnecessary NSAID use entails planetary health impacts (e.g., manufacturing, packaging, distribution, and waste) that scale when administered routinely at herd level. We did not conduct a systematic planetary health assessment, but NSAID courses given without clinical benefit add production and packaging footprints without offsetting clinical benefit when the sole aim is to prevent antimicrobial re-treatment. This reinforces avoiding routine co-administration for that purpose, while reserving NSAIDs for clear welfare indications (see Recommendation 5), used at the correct dose/duration with proper disposal and adherence to withdrawal periods.
• Implementation: This recommendation does not preclude NSAID use for welfare indications. When NSAIDs are used for pain/pyrexia, clinicians should follow label directions and withdrawal periods, and weigh case-by-case benefits and risks (see Recommendation 5).

### Certainty in the net effect (for preventing re-treatment)

For the specific outcome of preventing antimicrobial re-treatment, certainty is high. We did not downgrade for any GRADE domain: risk of bias (estimates consistent across RoB strata), inconsistency (I2 = 0% and effects uniformly within the prespecified trivial range), indirectness (the outcome precisely matches the narrow decision question; populations and interventions reflect field use), imprecision (95% CIs fully within the trivial range), or publication bias (funnel plot without concerning asymmetry). This differs from the broader NSAID systematic review certainty rating, where re-treatment was downgraded for indirectness as a surrogate for overall clinical cure and welfare outcomes; here the recommendation is deliberately limited to the outcome of re-treatment itself. As this recommendation is purposefully outcome-specific, it does not address welfare outcomes (analgesia, antipyresis, appetite/comfort), which are considered separately in Recommendation 5.

### Conclusion

• Given consistent, precise evidence that adding NSAIDs to antimicrobials does not reduce the need for antimicrobial re-treatment, and considering potential harms, burden, and costs, the panel recommends against NSAID co-administration for the specific purpose of preventing re-treatment. This recommendation does not address analgesia, fever reduction, or appetite/comfort; those welfare-related uses are considered separately (Recommendation 5).

### Recommendation 5

**In cattle with acute undifferentiated bovine respiratory disease, we make no recommendation for or against the use of NSAIDs for other outcomes (e.g., pain relief, reduction of pyrexia).**

*The certainty of the effect estimates for these outcomes is very low; any direction of effect is highly uncertain. The panel judged that current evidence is insufficient to identify subgroups most likely to benefit but agreed that treatment may be required to reduce apparent animal suffering. (100% agreement).*

Remark:

- Clinicians may consider NSAIDs in addition to antimicrobial treatment on a case-by-case basis for animals assessed to be in pain, balancing potential benefits and harms and adhering to label directions and withdrawal periods.

### Rationale for recommendation 5 (narrative summary of the evidence)

Across randomized trials, NSAID adjunct therapy often produced a faster early reduction in pyrexia (first 6–24 hours) and short-term improvement in clinical scores compared with antimicrobials alone (e.g., tulathromycin + ketoprofen vs tulathromycin alone; oxytetracycline + flunixin vs oxytetracycline; florfenicol + flunixin vs florfenicol; ceftiofur + NSAID vs ceftiofur) ( Scahill et al., 2026b). In several studies, these differences converged by 24–48 hours, and at 24 hours many groups were afebrile irrespective of NSAID use. Neutral findings were also reported (e.g., similar 24-hour temperature reduction with tilmicosin ± flunixin). Heterogeneous case definitions, antimicrobials, and time points, frequent allowance for rescue treatment after 3–5 days, and reporting of means/graphs without aligned variance precluded pooling of study results.

Evidence for longer-term welfare or performance benefits is inconsistent. In pre-weaned dairy calves, meloxicam adjunct therapy showed no benefit on clinical or lung ultrasonography scores or growth ( Ferree et al., 2023), whereas one older feedlot study reported higher average daily liveweight gain with oxytetracycline + meloxicam ( Friton et al., 2005), an isolated finding not reproduced in more recent trials. Overall, for welfare-related outcomes (analgesia/antipyresis/comfort), the evidence remains mixed, non-poolable, and of very low certainty. Clinically, NSAIDs may be considered case by case to alleviate apparent pain or high fever, following label directions and withdrawal periods, recognizing that they do not reduce antimicrobial re-treatment (see Recommendation 4). Full study details are summarized in the supplementary files in the NSAID systematic review ( Scahill et al., 2026b).

### Other decision criteria and considerations

• Values and preferences: The panel places high value on alleviating pain and distress and recognizes that many farmers and clinicians expect rapid comfort benefits. At the same time, when welfare benefits are uncertain or short-lived, the panel places value on minimizing avoidable drug use, costs, handling, withdrawal-time burdens, and planetary-health impacts.
• Harms: Adverse events were sparsely reported; one multi-country RCT noted some withdrawals due to enteritis without a clear pattern, but most studies reported no clinically important NSAID-related harms.
• Planetary health/AMR: While NSAIDs do not raise AMR concerns, avoidable herd-level use adds planetary-health burdens (manufacturing, packaging, distribution, waste); this reinforces reserving NSAIDs for clear welfare indications.
• Implementation: Potential for masking clinical deterioration: As NSAIDs reduce fever and relieve pain, they can transiently suppress clinical signs of worsening pneumonia (e.g., normalizing temperature or improving demeanour while lung pathology progresses). To avoid delayed antimicrobial change or rescue treatment, plan an early reassessment (e.g., at 24–48 hours) based on a complete clinical examination (temperature plus respiratory effort, auscultation/induced cough, appetite/attitude; use thoracic ultrasonography where available), rather than temperature alone. NSAID use for welfare indications should never replace timely review of antimicrobial response.

### Certainty in the net effect (welfare outcomes)

For analgesia/antipyresis/comfort, evidence remains very low certainty: short-term temperature/score improvements are inconsistently reported, often converge by 24–48 hours, and do not translate consistently into better lung ultrasonography, relapse, or growth. Reporting heterogeneity and allowance for rescue therapy precluded pooling of results. This supports a “no recommendation for or against”, with case-by-case clinical judgement required.

### Recommendation 6

**In cattle with acute undifferentiated bovine respiratory disease, we make no recommendation for or against using non-steroidal anti-inflammatory drugs (NSAIDs) as a substitute for antimicrobials.**

*The balance of benefits and harms is likely to vary substantially between individual animals and different herds, and the certainty of evidence is very low. Evidence is insufficient to define subgroups that would reliably benefit; therefore, an overall recommendation is not possible (100% agreement).*

Remarks:

- Any potential role for NSAIDs as a stand-alone option should be limited to carefully selected, mildly affected animals which can be closely monitored, with prompt initiation of antimicrobial treatment (rescue therapy) in case of clinical deterioration.
- Animals with moderate to severe disease, systemic illness, rapid clinical progression, or comorbidities should not have antimicrobials withheld.
- Decisions should be made on a case-by-case basis, considering animal welfare, antimicrobial stewardship, regulatory requirements, and withdrawal periods.

### Rationale for Recommendation 6

Summary of the evidence, benefits, and harms.

This question asks whether NSAIDs can be substituted for antimicrobials to treat acute, undifferentiated BRD. Evidence comes from two small RCTs in pre-weaned dairy calves in the UK that compared NSAID monotherapy (flunixin) ( Mahendran et al., 2017) with antimicrobial monotherapy (gamithromycin or florfenicol) ( Mahendran, 2020). Pooled results did not show a clinically meaningful advantage for NSAID monotherapy; if anything, they trended unfavorably for short-term re-treatment: RR 1.19 (95% CI 0.72–1.97), corresponding to 88 more re-treatments per 1000 animals (from 129 fewer to 448 more). Both trials used fever detection to trigger enrolment, and thoracic ultrasonography suggested little or no lung consolidation in many calves, indicating that populations likely included viral/upper respiratory presentations rather than established pneumonia. Across studies, NSAID-related adverse events were infrequently reported; however, the principal potential harm is undertreatment and delayed effective antimicrobial therapy in calves progressing to bacterial pneumonia. As NSAIDs reduce fever and discomfort, they might temporarily mask clinical deterioration and preclude timely antimicrobial change if used without close reassessment (see implementation notes).

### Other decision criteria and considerations

• Indirectness and feasibility: The trials were conducted in a narrow setting (pre-weaned UK dairy calves, fever-tag case finding and may not reflect typical field presentations across production systems. One study suggested a trend towards higher early re-treatment in the NSAID-only arm. Together with ethical and regulatory considerations in many jurisdictions (e.g., label indications for antimicrobials in pneumonia), this argues against generalizing NSAID monotherapy beyond carefully selected, closely monitored mild cases.
• Stewardship and planetary health: NSAIDs do not raise AMR concerns. That said, avoidable NSAID use does add planetary-health burdens (manufacturing, packaging, distribution, waste). The larger stewardship risk here is withholding needed antimicrobials in animals with bacterial pneumonia.
• Clinical experience: The panel members reported limited real-world experience using NSAID monotherapy across production systems, which increased uncertainty about values, acceptability, and feasibility in typical field practice.

### Implementation considerations (if monotherapy is contemplated)

• Selection: only mildly affected, closely monitored cases; avoid in dehydrated/hypovolemic calves or where pneumonia is suspected/confirmed (e.g., consolidation identified by thoracic ultrasound [TUS]).
• Monitoring: early reassessment at 24–48 hours using a comprehensive clinical examination (temperature, respiratory effort, cough, appetite/attitude; TUS where feasible); initiate or switch to antimicrobials promptly if not improving.

### Certainty in the net effect

Certainty is very low. We rated down for imprecision (the confidence interval spans multiple clinical thresholds in both directions), indirectness (re-treatment as a surrogate; narrow setting of pre-weaned UK dairy calves with fever-tag enrolment and limited consolidation), and study limitations/size (two small trials with different comparators). Taken together, these considerations support making no recommendation for or against NSAID monotherapy and emphasize early reassessment with predefined rescue antimicrobials when monotherapy is contemplated in carefully selected mild cases. The GRADE EtD Summary of Judgments ( Table 2) indicates very low certainty and an uncertain net effect for NSAID monotherapy versus antimicrobials, supporting no recommendation for or against.

## Conclusion

Given very uncertain and highly imprecise evidence, the panel makes no recommendation for or against using NSAIDs as a substitute for antimicrobials. Where NSAID monotherapy is contemplated, it should be limited to carefully selected, mildly affected animals with close monitoring and predefined rescue criteria and never used in animals with moderate to severe disease, systemic illness, rapid progression, or comorbidities. Prompt initiation of antimicrobials is warranted if clinical improvement is not seen on early reassessment (e.g., 24–48 hours). This stance complements recommendation 4 (NSAIDs do not reduce antimicrobial re-treatment when used as adjuncts) and recommendation 5 (case-by-case NSAID use for welfare indications).

### Recommendations for future research

Despite the widespread use of antimicrobials and NSAIDs for BRD, the certainty of evidence supporting many treatment decisions remains low or very low. Future research should prioritize large, pragmatic, multicenter randomized controlled trials using standardized BRD case definitions, clinically relevant outcomes, and harmonized follow-up periods to improve comparability across studies. Initiatives to develop a core outcome set for BRD studies are ongoing, which will improve reporting, data availability and quality for future meta-analyses.

Specific research priorities include:

1. Direct head-to-head comparisons between commonly used first-line antimicrobial classes, particularly in settings with differing *Mycoplasmopsis bovis* prevalence and AMR patterns.
2. Evaluation of individualized treatment strategies integrating clinical assessment, rapid diagnostics, thoracic ultrasonography, and antimicrobial susceptibility testing.
3. Studies assessing longer-term outcomes beyond short-term re-treatment, including mortality, relapse, chronic lung pathology, production performance, and animal welfare indicators.
4. Studies on determining the optimal therapy duration, in function of lesion severity and involved pathogen to identify a standard recommended therapy length for each category.
5. Improved characterization of antimicrobial-associated harms and AMR selection, including impacts on respiratory and gastrointestinal microbiota and resistomes.
6. Development and validation of clinically meaningful and standardized outcome measures for BRD trials, including consensus definitions of treatment failure and relapse.
7. High-quality trials evaluating NSAIDs for welfare-related outcomes such as pain, pyrexia, appetite, and recovery, including identification of subgroups most likely to benefit from this treatment.
8. Research evaluating the feasibility, safety, and stewardship implications of delayed antimicrobial strategies or NSAID-supported selective treatment approaches in mild BRD cases.
9. Implementation and adaptation studies assessing how guideline recommendations perform across different production systems, regulatory environments, and geographic regions using GRADE-ADOLOPMENT approaches.
10. Further exploration of precision medicine initiatives, individualizing therapy based on host and pathogen related factors, with the aim to minimize antimicrobial use.

### Limitations

Evidence base and publication bias: We restricted evidence to peer-reviewed RCTs and did not include grey literature. Although regulatory bodies publish assessment summaries (e.g., EMA veterinary EPARs; FDA/CVM FOI Summaries), complete clinical study reports and datasets are typically not publicly available, and unpublished industry trials are difficult to systematically identify. Given the lack of compulsory trial registration in veterinary medicine, publication/availability bias cannot be excluded.

Applicability and language: Most trials were conducted in Europe or North America and we applied an English-language restriction. Generalizability may therefore be limited in regions with different production systems, product authorizations/withdrawals, pathogen/AMR epidemiology, and practice patterns.

Outcomes and heterogeneity: Short-term “need for re-treatment” was used as a pragmatic surrogate for therapeutic failure; observation windows and allowances for rescue therapy varied, limiting attribution of longer-horizon outcomes (mortality, distant relapse, performance) to the index drug. Many trials were small and/or older, with heterogeneous case definitions, dosing/route, and follow-up intensity. Welfare outcomes for NSAIDs (analgesia, antipyresis, appetite/comfort) were inconsistently defined and not poolable.

Stakeholder input and EtD judgments: Stakeholder-derived clinical thresholds were obtained by convenience sampling predominantly from Europe and North America. Context domains in the Evidence-to-Decision frameworks (resources/cost-effectiveness, acceptability, feasibility, planetary-health implementation) reflect panel baseline judgments and were not supported by separate systematic reviews. Users should adapt the recommendations locally (ADOLOPMENT).

## Ethics and consent

Ethical approval and consent to participate were not required. This guideline synthesizes published, aggregate study data and panel judgments; no new animal or human experiments were conducted. Consent for publication: not applicable. The companion systematic reviews analyzed published, study-level data only and did not require ethics approval.

## Data Availability

No new primary data were generated for this guideline. Conclusions are based on summary effect estimates from published randomized controlled trials synthesized in two companion systematic reviews ( Scahill et al., 2026a, 2026b) and on panel Evidence-to-Decision (EtD) judgments. Open Science Framework (OSF): *ENOVAT BRD Guideline – Extended materials* ( Scahill, 2026), https://doi.org/10.17605/OSF.IO/3NVHY This project contains the following extended data:
• Supplementary File 1: Conflict of Interest (COI) forms. • Supplementary File 2: Stakeholder associations invited for consultation. • Supplementary File 3: Macrolide within-class rapid review, including methods, study-level extraction, forest plots, Supplementary Tables S1–S2, and sensitivity analysis forest plots for PICO 8. • Supplementary File 4: RIGHT checklist completed for this guideline. The RIGHT checklist is provided in Supplementary File 4 and is available at OSF: https://doi.org/10.17605/OSF.IO/3NVHY **License** All extended data are available under the terms of the Creative Commons Attribution 4.0 International (CC BY 4.0) license.
