# Supplementary material for: European Network for Optimization of Veterinary Antimicrobial Therapy (ENOVAT) guidelines for antimicrobial and non-steroidal anti-inflammatory drug use in bovine respiratory disease
Source: Open Res Eur. 2026 Aug 14;6:296. [Version 1] doi: 10.12688/openreseurope.24099.1 (PMC13601791; doi:10.12688/openreseurope.24099.1)
Supplement: Peer review history

# Reviewer report: Reviewer response for version 1

**DOI:** 10.21956/openreseurope.26088.r80057

**Published:** 2026-10-05

**Authors:** Michael D Apley

**Affiliations:** 1 Kansas State University, Manhattan, Kansas, USA

The paper is clearly written and methods and reasoning are transparent.  I recognize all of the work that has gone into the paper and thank the authors for their clarity and transparency.  I have some disagreements and concerns related to the totality of components involved in the final recommendations that lead me to ratings of “partly” for analysis and conclusions ratings.  This is not because I am unable to track the effect of these components, but that in my opinion the resulting recommendations will be cited without many considering the effect these components have had.     

 I have some strong concerns related to the conclusions that will just have to be accepted as differences of opinion as I evidently differ greatly from your group consensus.

1. I find the stakeholder-derived clinical thresholds to add yet one more layer of consensus-based subjectivity to the findings and remain puzzled as to what it actually adds to the analysis.  It results in dismissing legitimate differences as subjectively insignificant, e.g. tulathromycin/gamithromycin vs. tilmicosin (granted a P value of 0.07, but a fair value considering the big variation between study situations), and tetracyclines vs. fluroquinolones. I am troubled that significant differences between antimicrobial groups are dismissed based on yet another layer of subjective judgement. And I am frankly quite puzzled (as are apparently the authors) that a group of clinicians found a difference in first treatment response of anything less than 200/1000 as trivial.  This adds to the analysis the concept that retreatment of less than 200/1000 treated animals is an insignificant difference?  What about animal welfare, labor, cost, mortality?  When this value is so subjective and the authors feel the need to explain how it may or may not apply, what does it add to the paper other than an unnecessary inclusion which skews the final conclusions?     
2. This is your set of guidelines, and you can set the process up as you wish, and you set the process up transparently, but it appears to me that the rankings are basically converting human therapeutic importance rankings into risks.  This is where a difference in opinion exists in how far the precautionary principle should be inserted into the application of clinical data and making decisions on the optimal therapeutic approach for our patients.   Enough said, we just differ substantially in that approach. 

I understand you described the clinical thresholds in more detail in the antibiotic paper, but I suggest a little more detail about exactly what those numbers mean, as in 200 what?  The coverage of this concept in this paper is rather vague when first introduced as to the fact this concerns treatment failures.

 The authors do a good job of describing limitations of the analysis in the “Certainty in the net effect” section.

 Thank you for your additional within-class macrolide analysis.  It gets down to what one considers to be clinically significant.  I would view the tulathromycin/gamithromycin vs. tilmicosin responses quite differently as to existence of a difference and the triviality of the difference considering what it takes to capture and retreat a second treatment in the North American systems I work with, as well as considerations of animal welfare, labor, and cost.  I note the authors are transparent about these local conditions being taken into account and were very transparent as to the outcomes.  While according to your consensus clinical threshold criteria the macrolides are the same, I disagree, both from the presented data and also suggesting consideration of a spectrum difference among the macrolides used for bovine respiratory disease as discussed below.   

 One of the things considered in the paper is the difference in considerations when Mycoplasmopsis bovis might be present.  Penicillin is noted as an exclusion when this organism is suspected as a contributory pathogen, since we all know the lack of a cell wall precludes activity of the beta-lactams against Mycoplasma spp.  I suggest considering if tilmicosin also ends up on the exclusion list when Mycoplasmopsis bovis is considered a primary pathogen.  We are hampered by lack of standardized antimicrobial susceptibility testing methods and interpretive criteria for M. bovis, and we are well aware of the large impacts of small changes in media pH for these compounds, but I would make a case to consider tilmicosin as poorly active against M. bovis.  The in-vitro activity against several Mycoplasma species noted on the current North American Micotil label does not include M. bovis.   On the original label they actually noted the species with MICs (mcg/ml), these were M. dispar 0.097, M. bovirhinis 0.024, and M. bovoculi 0.048.  Note that M. bovis is not mentioned.  MIC values for the major respiratory pathogens on that same label were reported as 3.12 to 6.25 mcg/ml (M. haemolytica, P multocida, and H. somni).   Sulyok, et al., (BMC Vet Res, 2014) reported an MIC50 and MIC90 of 128 mcg/ml for tilmicosin against M. bovis.  Ayling, et al., (Vet Rec 2000) reported the same ranges.  Cai, et al (Can J vet Res, 2014) reported large differences between tilmicosin and tulathromycin for M. bovis MIC values.   A 2019 report by Klein, et al., (Vet Micro, 2019) showed what is arguably a quite different M. bovis distribution for tulathromycin and tilmicosin, with 119 of 232 M. bovis isolates having an MIC 50 and 90 of 64 mcg/ml for tilmicosin, the highest dilution tested.   Tulathromycin, which has Mycoplasmopsis bovis on the label, has M. bovis MIC values close to the other respiratory pathogens on the label.  Tilmicosin, which does not have M. bovis on the label, has a wide gap between the reported MIC values of label pathogens and that of M. bovis.  This is another component of my argument that macrolides are not a conveniently homogenous group.  At the least, I would suggest that these differences in relative MIC values for tilmicosin should at least be mentioned as a consideration.  Besides penicillin, the others in this paper not having M. bovis on the label (in U.S.) include tildipirosin, tilmicosin, oxytetracycline, and danofloxacin.  The only way to rule in these without a label claim for M. bovis is either to provide clinical data for this pathogen or to make an argument for similar M. bovis MIC values to the other labeled respiratory pathogens (with recognized caveats to this approach).  I make the argument tilmicosin should be ruled out.

 I would speculate that the support for oxytetracycline recommendation as a first line treatment in this paper when M. bovis is present is largely based on MIC spectrum analysis where M. bovis MIC values are close to other respiratory pathogens (I am not aware of a tetracycline class BRD label which includes M. bovis)?  Or was there consideration for evidence that M. bovis is in the spectrum for each antimicrobial, other than it is not for Penicillin?  Maybe I have missed an M. bovis label for oxytetracycline in another country, but I think you need to justify its inclusion for when M. bovis is present.    

**General comments:**

 I think this discussion also highlights a growing challenge in the published literature relating to conclusions drawn from the meta-analysis of available data sets.  There are multiple proprietary data sets being generated in large systems which are unavailable to an analysis such as this.  These data sets highlight the admonitions of the authors to consider these recommendations in light of local conditions.  The paragraph on “National and regional guideline adaptation” was certainly well received. 

 Overall, I found the NSAID section to be very well written with clear communication of reasons for decisions.

 The authors are completely transparent about methods, and a thorough evaluation of the paper allows the reader to understand the methods and discard some components if wished (the effect thresholds for me).  However, from my teaching and continuing education experience, complex study considerations are often reduced to a slide or two and I would opine that the presentations on this paper will be confined to the recommendation tables, which incorporate your interpretations of effect thresholds.  The same will be the fact for busy policy makers.  In the end, your effect thresholds and insistence that all macrolides are the same (that is, the ones we use for BRD, hopefully we all recognize that azithromycin is quite different in several aspects) results in overall, condensed conclusions which I, in my opinion, consider misleading as to the actual effects of the antimicrobials in BRD.  For example, using the effect threshold to conclude that the clinical effect of tetracyclines in BRD is equivalent to fluoroquinolones is a credibility crusher in my opinion.

Is the study design appropriate and does the work have academic merit?

Yes

Is the work clearly and accurately presented and does it cite the current literature?

Yes

If applicable, is the statistical analysis and its interpretation appropriate?

Partly

Are all the source data underlying the results available to ensure full reproducibility?

Yes

Are the conclusions drawn adequately supported by the results?

Partly

Are sufficient details of methods and analysis provided to allow replication by others?

Yes

Reviewer Expertise:

Clinical pharmacology with an emphasis on food animals, bovine respiratory disease, beef production medicine

I confirm that I have read this submission and believe that I have an appropriate level of expertise to confirm that it is of an acceptable scientific standard, however I have significant reservations, as outlined above.

# Reviewer report: Reviewer response for version 1

**DOI:** 10.21956/openreseurope.26088.r79328

**Published:** 2026-09-23

**Authors:** Naod Thomas Masebo

**Affiliations:** 1 Bologna University, Bologna, Italy

This paper is an important article which discusses the guidelines for responding to an important disease of cattle, which is bovine respiratory disease (BRD). BRD remains a challenge in the bovine production system in the EU; therefore, this guideline contains important information and steps for veterinary clinicians on how to respond to undifferentiated BRD in cattle.

 The guidance and recommendations provided in this guideline are very important for veterinary practitioners.

 The document is well written, and the recommendations are clear to implement.

 The methodology is presented and clearly written.

 The guidelines could be helpful material in other regions of the world to treat undifferentiated BRD in cattle as recommended in the guideline, customizing it to local conditions.

Is the study design appropriate and does the work have academic merit?

Yes

Is the work clearly and accurately presented and does it cite the current literature?

Yes

If applicable, is the statistical analysis and its interpretation appropriate?

Yes

Are all the source data underlying the results available to ensure full reproducibility?

Yes

Are the conclusions drawn adequately supported by the results?

Yes

Are sufficient details of methods and analysis provided to allow replication by others?

Yes

Reviewer Expertise:

Clinical medicine, Animal welfare, Animal behaviour, precision livestock farming

I confirm that I have read this submission and believe that I have an appropriate level of expertise to confirm that it is of an acceptable scientific standard.

# Reviewer report: Reviewer response for version 1

**DOI:** 10.21956/openreseurope.26088.r80054

**Published:** 2026-09-08

**Authors:** Ramon Armengol

**Affiliations:** 1 University of Lleida, Lleida, Spain

The present research study aims to provide simple and direct recommendations, achieved in a very reliable way in a complicated process, on the use of antibiotics and NSAIDs. Honestly, regarding the content and the work that the researchers have done, I have no major concerns. I congratulate the authors for the work done and I think it can be useful for cattle farms and practitioners, since as is clear, it is one of the bovine diseases that causes the greatest consumption of antimicrobials.

 However, I do have few minor comments/suggestions that I think should be considered before indexing. I detail them below and I hope they are understood, since I cannot detail lines of the text (the reviewable version does not have lines numbered):

- From GRADE, in methods (pg 2) till the final pages, authors must review ALL the abbreviations used and be clear and consistent. First time in the text, you explain what the abbreviation means and from then, only the capitals, please. Please, check the whole work, as examples: GRADE, WHO, M.bovis, PICO, TUS, NSAIDs...
- In page 3, I suggest including “should/could” in the sentence…”These ENOVAT SHOULD/COULD help vets and farmers…”
- In page 8, “Therapeutic failure (failure of clinical disease to resolve within 7 days…): Why 7 days? Please, explain, detail and/or add reference.
- At the end of Recommendation 5: Add a CONCLUSION, like in the other recommendations.

Is the study design appropriate and does the work have academic merit?

Yes

Is the work clearly and accurately presented and does it cite the current literature?

Yes

If applicable, is the statistical analysis and its interpretation appropriate?

I cannot comment. A qualified statistician is required.

Are all the source data underlying the results available to ensure full reproducibility?

Yes

Are the conclusions drawn adequately supported by the results?

Yes

Are sufficient details of methods and analysis provided to allow replication by others?

Yes

Reviewer Expertise:

Bovine clinics and health. Bovine ultrasonography. Biosecurity. Ruminant infectious diseases.

I confirm that I have read this submission and believe that I have an appropriate level of expertise to confirm that it is of an acceptable scientific standard.
